# Is imprinting the result of “friendly fire” by the host defense system?

**DOI:** 10.1371/journal.pgen.1008599

**Published:** 2020-04-09

**Authors:** Miroslava Ondičová, Rebecca J. Oakey, Colum P. Walsh

**Affiliations:** 1 School of Biomedical Sciences, Ulster University, Coleraine, Northern Ireland, United Kingdom; 2 Department of Medical & Molecular Genetics, King’s College London, Guy’s Hospital, London, United Kingdom; University of Pennsylvania, UNITED STATES

## Abstract

In 1993, Denise Barlow proposed that genomic imprinting might have arisen from a host defense mechanism designed to inactivate retrotransposons. Although there were few examples at hand, she suggested that there should be maternal-specific and paternal-specific factors involved, with cognate imprinting boxes that they recognized; furthermore, the system should build on conserved biochemical factors, including DNA methylation, and maternal control should predominate for imprints. Here, we revisit this hypothesis in the light of recent advances in our understanding of host defense and DNA methylation and in particular, the link with Krüppel-associated box–zinc finger (KRAB-ZF) proteins.

## Introduction

In 2017, the scientific community lost a pioneering epigeneticist Prof. Denise Barlow, who sadly passed away in October of that year. Denise was known for her careful and thoughtful experiments characterizing imprinted loci, particularly at the mouse insulin-like growth factor 2 receptor (*Igf2r*) gene, where her seminal work led to a number of ground-breaking insights for which she is well remembered. These include the first description of imprinting at this endogenous locus [1], the discrimination of primary and secondary control regions at imprints [2], and regulation of imprinted loci by long noncoding RNA (lncRNA) [3]. Some among us also remember her for her insightful leaps of intuition, including the prediction that an antisense mechanism could control many maternally methylated imprinted loci [4], that lncRNA would often be involved [5], and that transcriptional interference would be the means by which the lncRNA would prevent transcription of the paternally inherited allele of *Igf2r* [6]. Many of these were subsequently found to be true, in part, at least, because of the elegant work done by her and her team on the *Igf2r* locus [7,8].

Although some of her predictions could be confirmed in her lifetime, we are still accumulating evidence for others. In a particularly visionary short Perspective article for *Science* in 1993 [4], Denise drew on what was then very limited data to propose that genomic imprinting might have evolved from a host defense mechanism that utilized DNA methylation to silence invading viruses. This was quite extraordinary at the time, when it must be remembered that there were only a handful of imprinted genes known, that it was unclear how important DNA methylation was for imprinting, and that there was a real paucity of data on links between the two. Denise based her hypothesis partly on the work of Richard Chaillet and colleagues, who showed that the *TG*.*A* transgene, when inserted in the mouse genome, displayed features associated with imprinting [9], including DNA methylation on one allele and parental origin–specific transcription. The *TG*.*A* transgene contained retroviral sequences, and earlier experiments from Rudolph Jaenisch’s lab had shown that retroviruses introduced into the mouse genome prior to implantation almost always became heavily methylated by midgestation [10]. Tim Bestor, in a widely cited review a few years earlier [11], had proposed that retroviral features would attract methylation and that the primary function of DNA methylation in eukaryotes would be in host defense, mirroring that in prokaryotes, in which methylation forms part of the host restriction system. Denise now made an intellectual leap and proposed that features of the imprinting box in *Igf2r* would resemble a retrovirus and thus attract DNA methylation. Further, she proposed that imprinting may primarily occur in the maternal germ line, because maternally introduced methylation could cause either repression (*TG*.*A*) or expression (*Igf2r*). These were, at the time, daring suggestions, as the total number of imprinted genes was tiny, and the role of methylation in repression of even retroviral sequences was unclear, not to mention the dearth of information on how antisense repression might work! Nevertheless, as we will describe next, the intervening years have provided a wealth of evidence to shore up these predictions. Furthermore, recent work in the area has led to exciting advances in our understanding of the link between the processes of retroviral and imprint gene silencing.

## Proposed link between retrovirus silencing and imprinting

Denise’s 1993 Perspective article was based on studying the few endogenous imprinted genes known in mice at the time (*Igf2*, *Igf2r*, *H19*, small nuclear ribonucleoprotein polypeptide N [*Snrpn*]) as well as the behavior of some transgenes that had been introduced into the mouse genome by pronuclear microinjection and behaved in an imprinted fashion, in particular, said *TG*.*A* transgene. Based on these and earlier work from Azim Surani [12], she presented a model (with hand-drawn figure!) for imprinting: this must involve both a sequence element or “imprinting box” as well as an “imprinting factor” that binds it. Furthermore, these were likely to be sex specific, as paternally imprinted genes would need a different box and germ cell factor from maternally imprinted ones, with the factors being expressed in the germline. Based on these assumptions, she outlined what she concluded must be the 4 key properties of imprinted loci. They were that (1) interaction between the factor and box was reversible; (2) it should affect transcription; (3) the factor adds an erasable mark to the box during gametogenesis, which can be removed in the next generation’s gametes; and (4) the mark should be heritable on chromosomes during embryogenesis. As noted by others, the patterns of DNA methylation at imprinted loci and more generally, during embryogenesis were already known to be consistent with a role for this epigenetic mark in imprinting. However, the first DNA methyltransferase 1 (DNMT1) knockout mice had only just been generated, and confirmation of an effect on imprinted loci would not come until later that year. She nevertheless correctly predicted that *Igf2r* transcription would be turned off in the mutants, based on her lab’s analysis of the region, which was the putative imprinting box at the gene.

What, then, was the link with retroviruses? It was known from earlier work by Jähner and Jaenisch that new retroviruses introduced to the mouse embryo prior to implantation attracted DNA methylation [13], which was associated with their silencing. Transgenes without obvious retroviral features but with high cytosine-guanine (CG) contents such as the *TG*.*A* transgene also became methylated, although in that specific and rather unusual case, in a parent-of-origin-specific fashion. Denise proposed that the imprinting box in *Igf2r* and the whole transgene for *TG*.*A* might resemble a retroviral intruder and so attract methylation as part of a host defense system. “This implies,” she wrote, “that the origins of gene imprinting lie in an existing biochemical system that serves to neutralize foreign invading DNA.” Furthermore, as methylation in the female germline was likely sufficient to either activate (*Igf2r*) or repress (*TG*.*A*) imprinted genes, imprinting might be intrinsically tied to a host defense function in the mammalian oocyte, with a lesser and possibly distinct mechanism at play in the male germline.

## Imprinted genes as retrotransposons

Although bold in conception, concrete support for these proposals was initially slow in coming. It was not until almost a dozen imprinted genes had been identified in mice and enough was known about their control mechanisms that Wolf Reik and Jörn Walter could compile a table showing that, even though about half the genes were expressed paternally, almost all were nevertheless controlled by methylation in the oocyte [14]. Current figures estimate that there are approximately 100 confirmed imprinted loci in mice controlled by approximately 19 known imprint control regions (ICRs) [15,16]. All but 4 ICRs are controlled by maternal methylation, the few exceptions being those controlling *H19*, Ras protein specific guanine nucleotide releasing factor 1 (*Rasgrf1*), Delta-like 1 homolog (*Dlk1*), and G protein-coupled receptor 1 (*Gpr1*). Although this was strong support for a largely maternally determined system, clear evidence for retroviral features in these genes was harder to find. Some studies suggested that long interspersed nuclear element 1 (LINE-1) retrotransposons may be enriched in imprinted regions [17], but further work indicated that that was not the case [18]. Only a relatively small number of imprinted genes showed clear evidence of resembling retrotransposons or having been passively retrotransposed themselves (reviewed in [19]). There were, nevertheless, intriguing cases among these. The Retrotransposon gag like 1 (*Rtl1*) and Paternally expressed gene 10 (*Peg10*) genes resemble the sushi group of retrotransposons [20], and some other imprinted genes have been identified using bioinformatic screens for features associated with retrotransposition, such as absence of introns, insertion into multiexonic hosts, and the presence of CG-rich regions [21]. A recent insertion of an active intracisternal A particle (IAP) retrotransposon also caused imprinted-like behavior of the neighboring *Agouti* gene, driven by transcription from the IAP promoter [22]. DNA methylation at the IAP 5′ long terminal repeat (LTR) accompanied silencing of the promoter and reversion of the phenotype, prompting much interest around other potential metastable epialleles. It also prompted Denise and colleagues to examine other imprinted regions and propose that CG-rich repeated DNA elements may be linked to imprinting too [23]. Interestingly, these 2 components (that is, direct repeats and retrotransposon elements), are found at the *Rasgrf1* imprinted gene, and both are needed for its imprinting, although in this case, in the male germline (see next).

**Fig 1:**
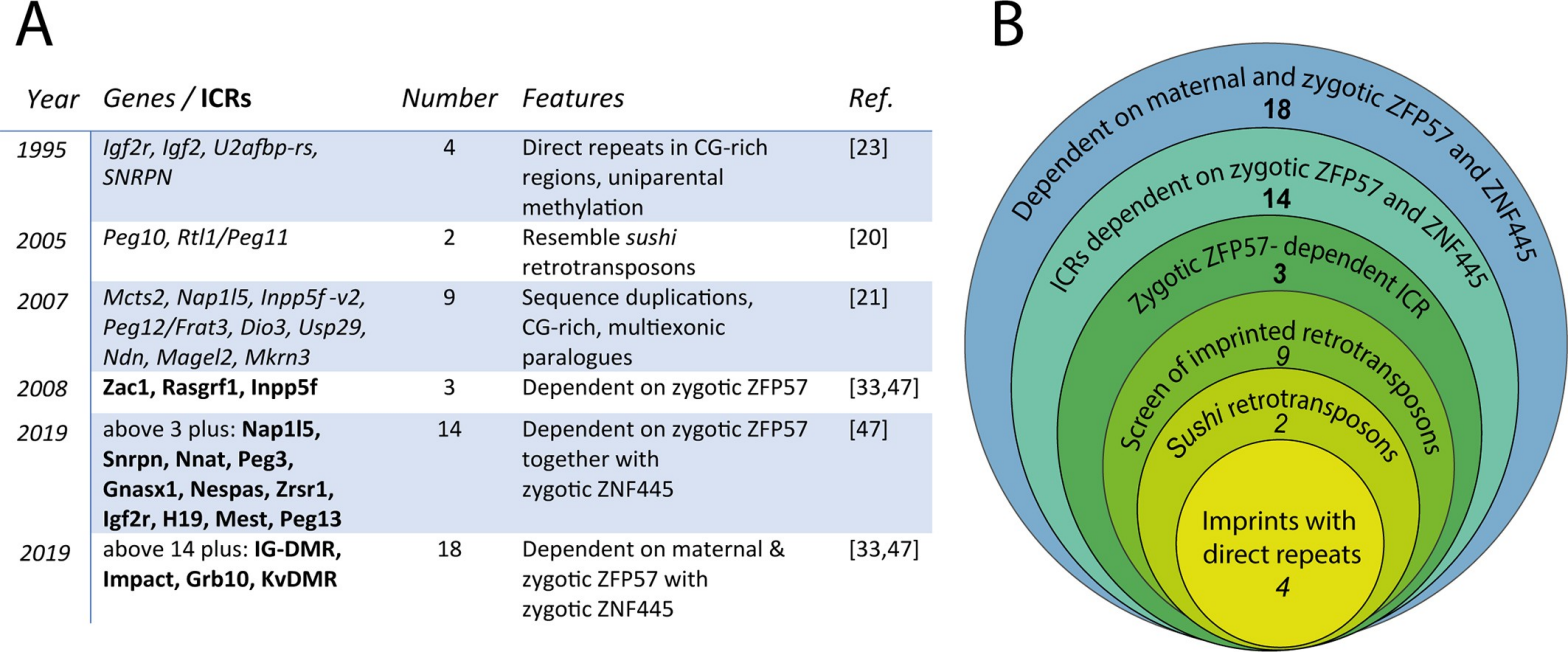
Only some of the maternally methylated imprints look like retrotransposons, but all are controlled by KRAB-ZF proteins. (A) Table showing progress in clarifying the transposon-imprinting link. Initial studies looked at individual genes (in italics) from the approximately 100 known; later work concentrated on ICRs (in bold) that regulate multiple genes: there are approximately 19 ICRs. Although a relatively small number of imprinted genes show clear evidence of retrotransposition, almost all are bound by, and many dependent on, zygotically expressed KRAB-ZFP57 for methylation maintenance. Recently, the Trono and Ferguson-Smith labs have demonstrated a clear role for a second KRAB-ZF protein (called ZNF445) in regulating a partially overlapping set of ICRs. If maternally expressed stores of ZFP57 are also depleted, even more ICRs are affected, thus covering 18/19 ICR. The one remaining ICR is Peg10, which is clearly related itself to the sushi retrotransposon class (row 2) and may be regulated by an as-yet-undiscovered KRAB-ZF protein. (B) Schematic summarizing the data shown in (A): the 3 smallest circles refer to genes, the larger to ICR, as above. Together, these studies clearly link all imprinted loci with host defense. CG, cytosine-guanine; ICR, imprint control region; KRAB-ZF, Krüppel-associated box–zinc finger; ZFP57, zinc finger protein 57; ZFN445, zinc finger 445, ZNF445.

On the whole, however, there was no clear evidence of a common retrotransposon signature associated with imprinted genes based on sequence evidence alone (Fig 1A), and the parent-of-origin effects at the *TG*.*A* transgene and the *Agouti* IAP insertions were too complex to really address her hypothesis. It wasn’t until the discovery of the maternal factor zinc finger protein 57 (ZFP57), which we will discuss next, that a clear common link was found between almost all imprinted loci and retrotransposon silencing.

## Maternal factors: DNMT3L and ZFP57

One of the other key predictions of Denise’s 1993 review was that maternal-specific imprinting factors would exist and that these should be of particular importance, given the dominant maternal role in imprinting. Early candidates for such a factor included the DNA methyltransferase DNMT1, which was found to have germline-specific transcripts with different translational potential [24], as well as the homologue DNA methyltransferase 3 like (DNMT3L), which is itself catalytically inactive but showed very germline-specific expression [25]. Although germline-specific knockouts of the former supported a limited role for the oocyte isoform of the protein in early development [26], the DNMT3L mutants showed a clearer maternal-effect loss of imprinting [27]. However, later work, which included the development of new techniques for looking at small pools of cells, showed conclusively that almost all sequence elements, not specifically imprints or retrotransposons, lose methylation in oocytes lacking maternal DNMT3L [28,29]. This suggested that DNMT3L acts more as a general cofactor for the de novo methyltransferases, and indeed, it has been shown by Ooi and colleagues [30] to act as a crucial bridge between histone and DNA modifications because it binds lysine 4 on histone 3, but only when unmethylated (H3K4me0), thus recruiting DNA methylation to inactive promoters. The germline factor primordial germ cell 7 (PGC7) is also important for maternal imprints, but its role is more general in preventing demethylation of the entire maternal genome in the very early embryo when the 2 pronuclei are still separate (well reviewed in [31]).

**Fig 2:**
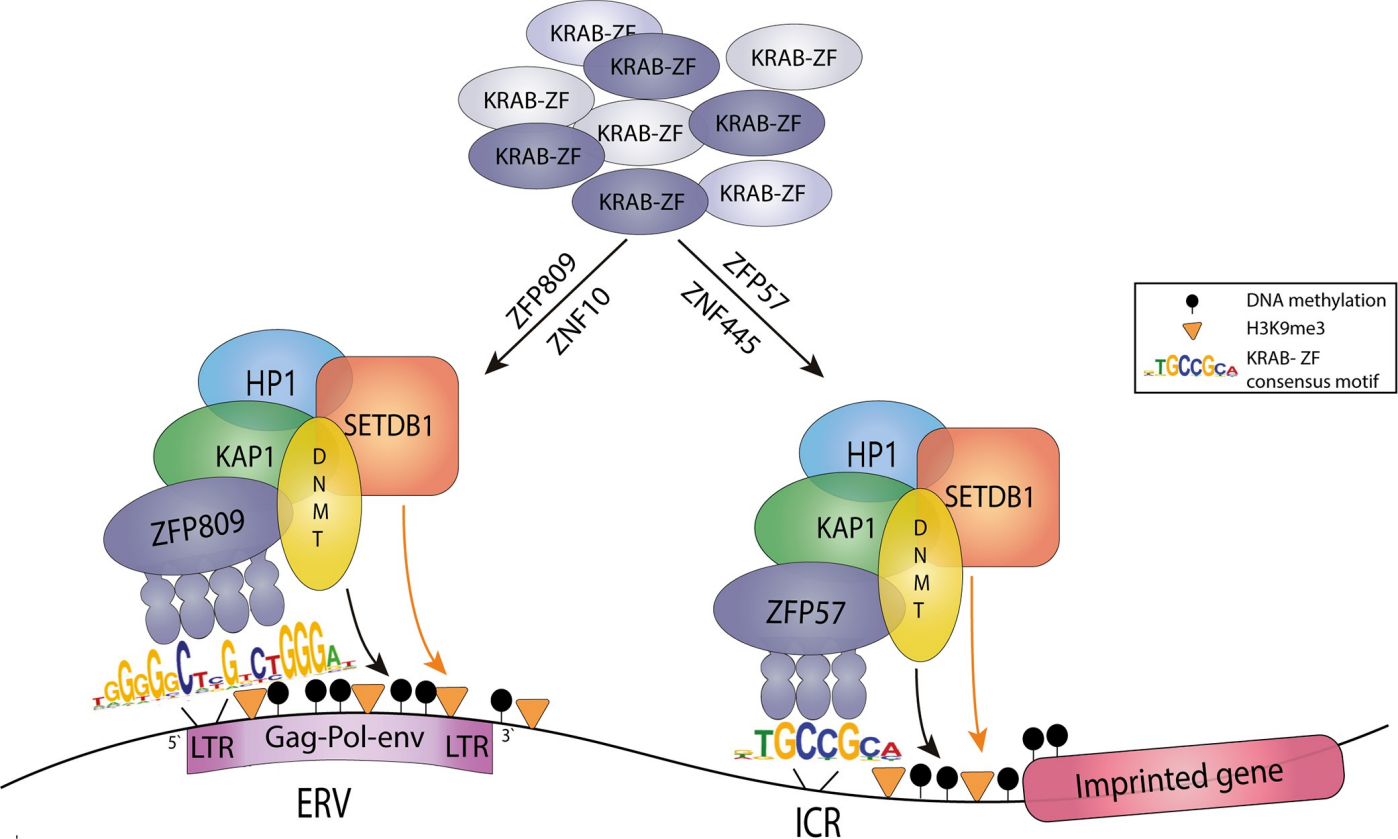
Identical cellular machinery is used to repress retrotransposons and imprints. From the large pool of KRAB-ZFPs known in mice, most target specific retrotransposon subfamilies, such as ZFP809 (shown at left [39]). which binds to a consensus sequence present on most PBS-pro-containing ERVs in mice, or ZNF10, which specifically targets HIV-1 in human [40]. Proteins involved in imprinting such as ZFP57 (shown at right) and ZNF445 appear to bind consensus sequences that are found at some ERVs but also at all ICRs. In both situations, the KRAB box recruits the transcriptional corepressor KAP1, which can mediate transcriptional shut-down in the short term in ES cells and early embryo through HP1 and SETDB1-mediated H3K9me3 deposition and in the longer term, postimplantation by recruiting DNMTs to methylate the DNA. DMMT, DNA methyltransferase; ERV, endogenous retrovirus; ES, embryonic stem; HIV-1, human immunodeficiency 1; HP1, Heterochromatin protein 1; IAP, intracisternal A particle; ICR, imprint control region; KAP1, KRAB-associated protein 1; KRAB-ZF, Krüppel-associated box–zinc finger; LTR, long terminal repeat; PBS-pro, primer binding site-proline; SETDB1, SET domain bifurcated 1; ZNF, zinc finger; ZFP, zinc finger protein.

The other 2, more intriguing factors implicated in maternal imprint establishment and maintenance form part of a single biochemical complex: these are KAP1 (KRAB-associated protein 1) and ZFP57. Although the effects of KAP1 deletion specifically on imprints was somewhat muddied by a severe but variable phenotype in offspring [32], there was clearer evidence of effects on a subset of imprinted genes in ZFP57 mutants [33]. The reason these 2 factors are of particular interest is that they form part of a larger system in the cell whose primary role appears to be the recognition and inactivation of retrotransposable elements [34–37]. KAP1 is a transcriptional corepressor that binds to the KRAB box present on Krüppel-associated box–zinc finger (KRAB-ZF) proteins and initiates transcriptional shut-down of the adjacent sequences by recruiting the histone methyltransferase SET domain bifurcated 1 (SETDB1), which adds the repressive mark H3K9me3, as well as DNA methyltransferases and the heterochromatin protein 1 (HP1) protein (Fig 2). Targeting of the sequences to be repressed is carried out by the KRAB-ZF family of proteins, which is coded for by a rapidly evolving gene family [34], likely reflecting the need to adapt to the emergence of new endogenous retroviruses (ERVs) that arise through natural evolutionary processes. Elegant work by the Trono lab has found a large number of KRAB-ZF genes are present in the mouse genome and the youngest of these appear to target the most newly-arisen ERVs, such as members of the IAP family [38], known to account for many de novo mutations through insertional mutagenesis in mouse (such as the mutation at *Agouti* mentioned previously). Thus, as novel ERVs arise, there is selection for new KRAB-ZF proteins to evolve with a binding domain that can recognize a key sequence element in the retrovirus.

The ZFP57 protein appears to be a divergent member of this KRAB-ZF protein family that has evolved to bind a specific sequence TGCCGC [41]. This sequence is present in nearly all known ICRs, thus acting as the imprinting box. Binding studies have confirmed, however, that the protein binds to many IAP elements and some other retrotransposon LTRs in the mouse genome too [42]. So, although the ICRs themselves do not bear any great resemblance to transposons as Denise originally suggested, the key recognition sequence has been derived using a mechanism that normally acts to recognize new transposons. Thus, a possible scenario is that this particular KRAB-ZF protein arose to combat a novel ERV containing the target sequence but the adjacent endogenous genes, or ones that happen to have a similar sequence, may have come under the control of the KRAB-ZF/KAP/DNMT system in a “friendly fire” type of scenario. Of course, in cases in which this led to benefits for the organism, for example, in balancing parental contributions to the embryo in the classic parental conflict scenario for imprinting [43], there was likely adaptive pressure to retain this form of transcriptional control. In other cases, selective pressure may not have been sufficiently large to either fix or remove the control mechanism, which might go some way to explaining why it is hard to fit all imprinted genes into any clear functional group and why there is such variation among tissues, stages, and populations in imprinting status for many genes [44].

Binding studies and knockouts have established that ZFP57 regulates many imprinted genes in mice [41,42]. Indeed, there is also a *Zfp57* orthologue in human, which, though divergent in structure, retains the recognition sequence and appears to be functionally required for imprinting, as mutations in the gene are found in a human syndrome characterized by a variable loss in imprinting, although the effects were not strong [45]. However it is, strikingly, 1 of only 2 loci known to cause multilocus imprinting defects in human, the only 1 that is a DNA-binding factor (the other is NLR family pyrin domain containing 2 [*NLRP2*]), and the only gene showing some conservation from human to mouse giving demonstrable alterations in methylation at ICRs when mutated (reviewed in [46]). Nevertheless, there remained a “gap,” i.e., genes that did not appear to be affected by mutations in the protein, both in mouse and human. A reasonable prediction from the aforementioned model was that there may be other KRAB-ZF proteins that regulated the remaining genes. Based on this assumption, Ferguson-Smith, Trono, and colleagues recently identified another family member zinc finger protein 445 (ZNF445), which bound to and regulated an overlapping set of ICRs [47], leaving only 1 ICR not bound either in human or mouse by these 2 KRAB-ZF proteins (Fig 1A): however, as this was the *Peg10* ICR, which is clearly a sushi-class retrotransposon, it may well be regulated by another KRAB-ZF protein that has not yet been uncovered. Interestingly, a role for the KRAB-ZF protein ZFP568 in regulating the placenta-specific promoter P0 of *IGF2* in mouse has also recently been shown by Yang and colleagues, further linking this class of transcriptional regulators with imprinted loci [48].

## Maternal versus paternal factors: KRAB-ZF proteins versus PIWI-interacting RNA

A key feature of imprinted repression mediated by KRAB-ZFPs is that it involves the female germline, with repression set up or maintained by factors expressed in oocyte. ERV repression driven by the larger KRAB-ZF protein family appears to reflect a system that is important for shielding the oocyte and early embryo from the overwhelmingly negative effects of these genomic parasites. Interestingly, although ZFP57 and ZNF445 are required for maintaining methylation on all ICRs, both maternal and paternal, in the early embryo, only a limited number of ICRs fail to establish methylation in *Zfp57* homozygous mutant oocytes [33]: thus, there may be other KRAB-ZF proteins, or indeed other factors, at work during oogenesis. What, then, of the male germline? It appears that during spermatogenesis, a similar host defense function is carried out by a different system involving PIWI-interacting RNA (piRNA). Much like in the CRISPR-Cas9 system, which is so widely used in biotechnology now, there is a recognition and an execution component to the system. The piRNA are small RNA transcribed from loci containing a battery of different ones, acting as a cellular memory for invading repetitive sequences by retaining copies of snippets of the offending element [49]. The piRNA interact with P-element induced wimpy testis (PIWI) proteins, which are transcribed in the male germline [50] to bind to and destroy any RNA with matches [49].

A clue as to the involvement of this system in regulating imprints as well as ERVs came from a key paper in *Nature* by Bourc’his and Bestor, who showed that DNMT3L deficiency in the male germline resulted in massive demethylation and transcriptional derepression of retrotransposons [51]. Unlike *Dnmt1* [51,52] or KRAB-ZF mutants, this affected both LTR (IAP) and non-LTR (LINE1) elements. Subsequent work showed that DNA methylation acted downstream of the piRNA system for repression of ERVs [53], serving, perhaps, as a mechanism for shutting down production of these elements at the source. The importance of this system was underscored by the discovery of a novel DNMT homologue DNMT3C [54], which is required for male fertility and specific to the murid lineage of rodents [54,55], the latter property possibly reflecting an increased ERV load in this order. Furthering the link between methylation, imprinting, and retrotransposon repression, Sasaki and colleagues went on to show that piRNA-induced DNA methylation is what drives the paternal imprinting of the mouse *Rasgrf1* gene [56], with the direct repeats identified by Denise playing an important role. Although generalizations are more difficult in the case of the male germline because of the paucity of examples (a mere 4 ICRs, with the other 3 not showing any clear dependence on the piRNA system as yet), it is nevertheless a striking case. The piRNA pathway is active in female mice but does not appear to be required for retrotransposon suppression because of a very active RNA interference (RNAi) pathway utilizing oocyte-specific Dicer (Dicer^O^) [57]. Whether the piRNA pathway is important in other mammals or in females of any species is currently unknown.

## Conclusions

Although much remains to be learned regarding the mechanisms and evolutionary pressures that have given rise to imprinting, and no doubt many imprinted genes will fail to conform to this model, it is nevertheless striking to see the parallels between Denise’s predictions [4] and what we currently know. Thus, we do indeed have (1) different maternal and paternal imprinting factors (KRAB-ZF proteins versus piRNA) as well as (2) imprinting boxes (ERV-derived ZF motif versus random ERV sequence), with (3) the presence of “foreign invading DNA” [4] the key sequence element, and (4) host defense mechanisms (SETDB1/DNMT and PIWI/DNMT) as the “existing biochemical system that serves to neutralize” it. Would that we all had such perspicacity!
